# Prevalence of Malnutrition in Patients with Liver Cirrhosis in A Tertiary Care Hospital

**DOI:** 10.31729/jnma.4533

**Published:** 2019-08-31

**Authors:** Dibas Khadka, Binod Karki, Suresh Thapa, Ajit Khanal, Ramila Shrestha, Shital Bhandary, Bidhan Nidhi Paudel

**Affiliations:** 1 Department of Medicine, National Academy of Medical Sciences, Kathmandu, Nepal; 2 School of Public Health, Patan Academy of Medical Sciences, Lalitpur, Nepal

**Keywords:** *cirrhosis*, *malnutrition*, *nutritional assessment*

## Abstract

**Introduction:**

Cirrhosis of liver is a progressively deteriorating disease. Medical management consist of treatment and prevention of complications. Nutritional intervention to improve nutritional status of the malnourished patient has favorable impact on prognosis. Traditional measures of nutritional assessment like Body Mass Index and waist circumference are inaccurate. This study was conducted to study the prevalence of malnutrition in cirrhotic patients.

**Methods:**

This was a descriptive cross-sectional study conducted at National Academy of Medical Sciences, Bir Hospital, Kathmandu, Nepal. The study included 60 cirrhotic patients from outpatient department. Subjective Global Assessment was performed for all participants and level of malnutrition recorded. Height, weight, waist circumference, body mass index were measured and recorded.

**Results:**

Among the cirrhotic patients, malnutrition was detected in 46 (76.66%) [68.38 to 85.94 at Confidence Interval 95%]. Out of 46 patients who were malnourished, 20 (43.47%) had mild to moderate malnutrition whereas 26 (56.53%) had severe malnutrition. The most common cause of cirrhosis was alcohol. The mean body mass index and mean waist circumference were within normal limits.

**Conclusions:**

Malnutrition is very common among cirrhotic patients and its prevalence increased from Child A to Child C status.

## INTRODUCTION

Cirrhosis is a late stage of progressive hepatic fibrosis characterized by distortion of the hepatic architecture and the formation of regenerative nodules. In Nepal, alcohol is the most common cause of cirrhosis followed by chronic viral hepatitis.^1^ Malnutrition has been reported in 50 to 100 percent of patients with decompensated cirrhosis and at least 20 percent of patients with compensated cirrhosis.^2–5^

Malnourished cirrhosis patients have higher rates of increased rates of septic complications, poorer quality of life, and a reduced life span when compared those with no malnutrition.^6,7^ Data on malnutrition in cirrhosis is emerging in recent years. There is paucity of data on malnutrition in Cirrhosis in Nepal.

This study has been conducted to identify the prevalence of malnutrition in patients with cirrhosis by using Subjective Global Assessment (SGA) Tool.

## METHODS

This was a descriptive cross-sectional study conducted at National Academy of Medical Sciences, Bir Hospital, Kathmandu, Nepal from December 2018 to May 2019. Ethical approval was taken from institutional review board. Informed consent for participation was obtained from all the participants. Patients attending outpatient department of Gastroenterology Unit and Hepatology Unit were included in the study. Cirrhosis of liver was diagnosed based on clinical, biochemical, and radiological findings suggestive of cirrhosis. Convenience sampling was used to collect data. Sample size was determined by using following formula:

n = Z^2^ × (pxq)/d^2^

=1.96 × 0.9 ×0.1 / 0.082

= 54

Here,

n = sample size
p = prevalence, 90% (educated guess)
q = 1-p
d = margin of error, 8%
Z = 1.96 at 95% CI

Taking non-response rate of 10%, calculated sample size was 60.

Nutritional assessment for detection of malnutrition was done by Subjective Global Assessment. After the assessment, patients were grouped in one of the three groups: well nourished, mild/moderately malnourished and severely malnourished. Patients with Hepatic Encephalopathy, Variceal Bleeding, and Spontaneous Bacterial Peritonitis were excluded. We excluded patients with uncontrolled diabetes mellitus, acquired immunodeficiency syndrome, tuberculosis, chronic renal failure, hepatocellular carcinoma or any malignancy were excluded. Detailed history and physical examination findings data were noted from the participants. Complete blood count, renal function test, liver function test, abdominal ultrasonography, prothrombin time, INR level data were collected.

Nutritional assessment was done through clinical and anthropometric measurements. Body Mass Index (BMI) was calculated by dividing body weight in kilogram divided by height in meter squared. Height was recorded in standing position, weight was recorded with a generic digital weighing scale. Waist circumference was measured with a measuring tape, midway between lower costal margin and highest point on iliac crest.

Data analysis was done using Microsoft Excel. Descriptive statistics were used and data are presented as mean (standard deviation) for continuous variables or median and ranges as appropriate. Percentages are reported for categorical variables.

## RESULTS

Among the cirrhotic patients 46 (76.66%) were found to have malnutrition as per SGA evaluation at the confidence interval of 95% (68.38 to 85.94). Out of 46 patients who were malnourished, 20 (43.47%) had mild to moderate malnutrition whereas 26 (56.53%) had severe malnutrition. Severe malnutrition was not detected in patient with Child A cirrhosis.

The study included 60 patients with cirrhosis of liver. Base line characteristics of the participants (Table 1).

**Table 1:** Baseline characteristics of participants.

Parameters	Values
Age years	
Male Mean ± SD^*^	48.10±11.01
Female Mean ± SD	44.8±12.5
Male n (%)	40 (66.66)
Mean weight kg ± SD	58.13±13.38
Median height meter (IQR^†^)	1.58 (1.49±1.63)
Median BMI^‡^ kg/m^2^	23.64 (20.41-26.59)
Mean waist circumference (cm) ± SD	91.67±10.34
SGA^§^ Class A n (%)	14 (23.33)
SGA Class B n (%)	20 (33.33)
SGA Class C n (%)	26 (43.33)

* Standard deviation

† Interquartile range

‡ Body Mass Index

§ Subjective Global Assessment

**Figure 1:**
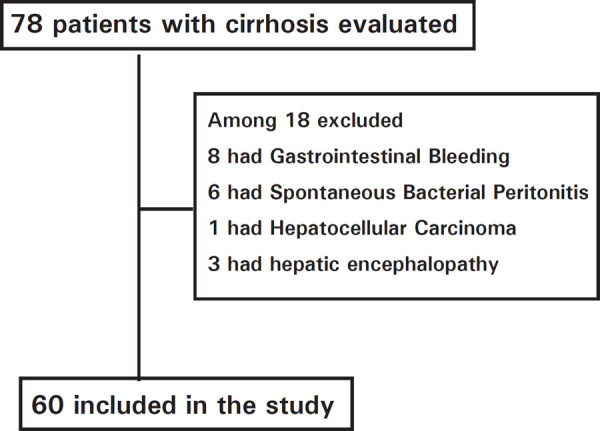
Enrollment of participants.

Prevalence of malnutrition increased with increasing class of Child Pugh classification of cirrhosis. All patients with Child C cirrhosis were malnourished. Six (27.27%) out of 22 patients in Child C cirrhosis had mild to moderate malnourishment while remaining 16 (72.72%) had severe malnutrition. Majority of patients had alcoholic cirrhosis. Distribution of causes of cirrhosis (Figure 1).

**Figure 2:**
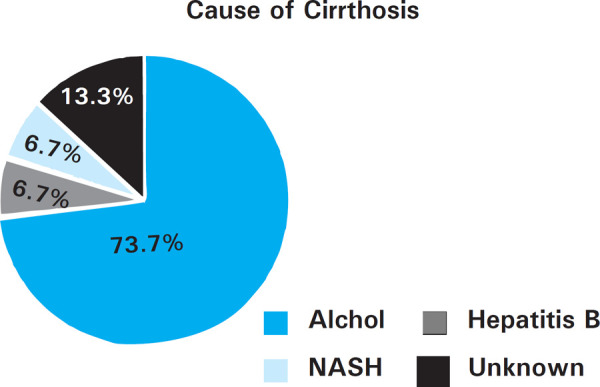
Cause of cirrhosis.

Blood investigations were performed in patients with cirrhosis (Table 2).

**Table 2:** Laboratory Parameters of Participants.

Parameters	Values
Mean hemoglobin gm/dl ± SD^*^	10.73±2.19
Albumin gm/dl ± SD	2.73±0.62
Serum creatinine mg/dl ± SD	0.84±0.25
Serum sodium mEq/l ± SD	134.53±4.50
Median Bilirubin mg/dl (IQR^†^)	2.10 (1.05-5.75)
INR^‡^ (IQR)	1.30 (1.19-1.52)
Median duration of abstinence weeks (IQR)	24 (7.5-52)
Pedal edema n (%)	46 (76.7)
Ascites n (%)	42 (70)
Bleeder n (%)	20 (33.3)
Hepatic encephalopathy n (%)	6 (10)
Varices n (%)	52 (86.7)
Diabetes n (%)	6 (10%)
Mean MELD^§^ Na ± SD	17.23 ±7.06
CTP^∥^ A/B/C n (%)	16 (26.66)/22 (36.66)/22 (36.66)

* Standard deviation

† Interquartile range

‡ International Normalised Ratio

§ Model for End Stage Liver Disease

∥ Child Turcotte Pugh

**Table 3:** Prevalence of malnutrition in different Child Pugh Cirrhosis.

	Malnutrition as per SGA*(%)
Child A n = 16	6 (37.5)
Child B n = 22	18 (81.81)
Child C n=22	22 (100)

Prevalence of malnutrition is greater among Child C than Child A and B (Table 4).

## DISCUSSION

Malnutrition in cirrhosis has been shown to be associated with adverse outcomes. In this study we utilized Subjective Global Assessment and other anthropometric measurements to assess the nutritional status of the patients. Subjective Global Assessment has been established as a reliable tool to detect malnutrition. The assessment was done as described by Detsky et al. The tool contains questions and physical examination. Questions are about weight change, dietary intake and functional capacity.^8^ Physical examination include assessment of subcutaneous fat, muscle wasting, edema and ascites.

Overall three fourth of patients were malnourished and severity of malnutrition increased from Child A to Child C Cirrhosis. In a study by Ciocîrlan et al, 100 patients with cirrhosis were evaluated.^9^ Around 66% of total patients were malnourished (SGA B or C). Among patients with Child C cirrhosis 93.54% had malnutrition. Sharma et al demonstrated that 56% of patients with cirrhosis had malnutrition as per SGA.^10^ In Child A Cirrhosis malnutrition was seen in 43.9% which is similar to this study. In Child B Cirrhosis and Child C Cirrhosis, 43.90% and 72.04% respectively had malnutrition.

Previously used parameters to assess nutritional status do not perform well in patients with cirrhosis. In the study by Ciocîrlan et al, all patients with cirrhosis had BMI in normal range.^9^ In our study, the median BMI in cirrhotic patients was within normal limit. In a population based study of individuals aged 15 to 69 years from Nepal, mean BMI was found to be 22.4 kg/m^2^. Mean waist circumference among males was 79.8 cm and in females it was 76.7 cm.^11^ Due to presence of ascites, patients were found to have higher waist circumferences. These factors make traditional measurements imprecise for assessing the nutritional status.

Vieira et al, in a study of 78 patients with cirrhosis, showed that SGA detected malnutrition in 61.5% of patients whereas BMI detected malnutrition in only 16.7% of cases.^12^ These findings demonstrate the limitation of BMI.

Hand Grip Strength is a newer measure of nutritional status. Ciocîrlan et al concluded that hand grip strength (HGS) and SGA were fair predictors of disease severity and 6 months survival in cirrhotic patients. HGS measurement evaluate the muscle activity of the dominant arm.^9^ Alvares-da-Silva et al evaluated 50 out patients with Cirrhosis.^13^ Majority of the patients were in Child A Cirrhosis. HGS was found to have better accuracy to detect malnutrition. HGS also predicted development of liver related adverse outcomes when patients were followed for 1 year. Limitations of this study include single center study and cirrhosis diagnosis without liver biopsy. Majority of participants had alcoholic cirrhosis which may not be true in other populations. Being a tertiary center, referred patients are likely to have severe disease than those presenting at other centers.

## CONCLUSIONS

Patients with liver cirrhosis have limited therapeutic options to prevent progressive deterioration. Nutritional status of the patient is commonly overlooked. With over three fourth of patients having malnutrition, proper nutritional intervention to treat malnutrition leads to better outcome.

Nutritional assessment should be an integral part of patient evaluation in patients with cirrhosis. Subjective Global Assessment tool is a reliable method to detect malnutrition.

## Conflict of Interest:

**None.**
